# Optimum protein requirement of juvenile orange-spotted grouper (*Epinephelus coioides*)

**DOI:** 10.1038/s41598-021-85641-4

**Published:** 2021-03-18

**Authors:** Xiaobo Yan, Junjiang Yang, Xiaohui Dong, Beiping Tan, Shuang Zhang, Shuyan Chi, QihuiYang, Hongyu Liu, Yuanzhi Yang

**Affiliations:** 1 Laboratory of Aquatic Nutrition and Feed, College of Fisheries, Guangdong Ocean University, Zhanjiang, 524088 People’s Republic of China; 2 Aquatic Animals Precision Nutrition and High Efficiency Feed Engineering Research Center of Guangdong Province, Zhanjiang, 524088 People’s Republic of China; 3 Key Laboratory of Aquatic, Livestock and Poultry Feed Science and Technology in South China, Ministry of Agriculture, Zhanjiang, 524000 Guangdong People’s Republic of China

**Keywords:** Physiology, Zoology, Animal physiology, Ichthyology

## Abstract

The purpose of subject was to explore the optimum protein requirement of juvenile grouper (*Epinephelus coioides*). In the test, 450 juveniles with an average weight (10.02 ± 0.22) g were randomly divided into six groups with triplicate, and were fed with 350, 400, 450, 500, 550 and 600 g/kg iso-lipid test diet twice 1 day for 8 weeks, respectively. The results showed that: (1) With the increase of protein level, the body weight gain rate and specific growth rate first increased and then reduced, while the feed coefficient rate first decreased and then increased, while the protein efficiency significantly decreased (*P* < 0.05). (2) With the increase of protein level, the condition factor, hepaticsomatic index and visceralsomatic index significantly reduced (*P* < 0.05). (3) With the increase of protein level, the crude protein content of whole fish and muscle gradually increased, while the crude lipid content gradually decreased. (4) High-protein diet (550–600 g/kg) significantly increased the plasma total protein content and decreased the triglyceride content of orange-spotted grouper (*P* < 0.05). (5) Compared with the 350 g/kg group, 500, 550, 600 g/kg groups significantly increased the activities of glutamic-pyruvic transaminase and glutamic oxaloacetic transaminase in liver (*P* < 0.05). (6) With the increase of protein level, the protease activity of intestine first increased and then decreased, and reached the maximum at the protein level of 500 g/kg, while lipase and amylase decreased significantly (*P* < 0.05). (7) The activities of acid phosphatase, superoxide dismutase and lysozyme in liver increased first and then decreased with the increase of protein level, and reached the maximum in the 400 g/kg protein group. According to the analysis specific growth rate, the optimum protein level of juvenile orange-spotted grouper is 521.84 g/kg.

## Introduction

Protein is one of the essential nutrients for all aquatic animal tissues and organs, as well as organic structure and function, accounting for 65–75% of the total dry matter of fish^1^. If the absorption of protein is lower than physiological needs, the growth of fish will slow down, stop or even lead to weight loss^2–4^, the decisive factor of fish growth speed is the content of protein in the body, because the growth of fish cannot be separated from the accumulation of protein in the body^5^; while the feed protein content exceeds the demand of the fish, only part of the feed protein is used for the synthesis of the fish body protein, and the rest enters the metabolic link, resulting in the increase of ammonia nitrogen excretion, resulting in the pollution of the aquaculture water environment^6,7^. It has been reported that the utilization of diet protein is influenced by the digestive enzymes activities^8^, and the deposition or accumulation of fish protein is regulated by the diet protein level^9^; moreover, protein not only plays an important role in holding normal vital movement, but also affects the physiological metabolism and immunity of fish^10,11^. Consequently, to ensure growth and cut the cost, and take into account the health of fish, breeding benefits and environmental protection, the protein content of feed needs to be kept at an appropriate level.

Orange-spotted grouper (*Epiephelus coioides*), belonging to Perciformes, Serranidae, is widespread in the southeast coast of China. It’s famous for superior growth rate, better adaptation environment ability, low mortality, strong resistance to adversity, fresh and tender meat and high nutritional value, it is welcomed by farmers and consumers^12,13^. At present, scholars have carried out a great deal of meaningful research about protein requirements of grouper. For example, the protein requirement of malabar grouper (*Epinephelus malabaricus*) is between 440 and 560 g/kg^14,15^, polka dot grouper (*cromolipetes altivelis*) is 440 g/kg^16^, and *centroprostis striata* is between 456.2 and 468.6 g/kg^17^. The protein requirement of grouper varies with varieties, specifications, protein sources, feeding systems and feeding conditions, but according to the data, the protein demand of grouper to maintain normal vital signs is 440–550 g/kg^18,19^. Previously, we have studied the protein requirements of grow-out^20^ and large-size orange-spotted grouper^21^, and there have been a report about protein requirement of juvenile orange-spotted grouper, but it is only limited to the study of growth performance^22^. Thus, present study aimed to explore the influence of protein level on growth performance, physiological biochemistry and immune index of juvenile orange-spotted grouper, so as to offer basis for accurate nutrition of juvenile orange-spotted grouper.

## Result

### Growth performance and morphological indices

**Figure 1:**
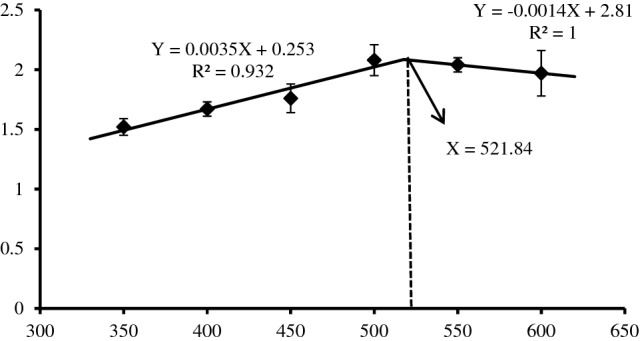
Relationship between dietary protein level and specific growth rate of juvenile orange-spotted grouper fed the experimental diets.

The weight gain rate (WGR) and specific growth rate (SGR) of grouper increased first and then decreased with the increase of dietary protein level (Table 1). When protein level was 500 g/kg group WGR and SGR got the maximum and was significantly higher than that in 350 g/kg and 400 g/kg groups (*P* < 0.05). At this time, protein level had no effect on survival rate (SR) (*P* > 0.05). With the increase of protein level, feed conversion ratio (FCR) first reduced and then up-regulated, just in the opposite state, and other groups higher obviously than the lowest value in 500 g/kg group, except for 550 g/kg group (*P* < 0.05). However, protein efficiency ratio (PER) lessened obviously with the increase of protein level (*P* < 0.05). According to the line chart analysis with SGR as the evaluation index, the optimal protein requirement of juvenile orange-spotted grouper was 521.84 g/kg (Fig. 1). The condition factor (CF), visceralsomatic index (VSI) and hepatosomatic index (HSI) of orange-spotted grouper reduced significantly with the increase of dietary protein level (*P* < 0.05) (Table 2).

**Table 1:** Effect of dietary protein level on growth performance of juvenile orange-spotted grouper.

Parameters	Dietary protein levels (g/kg)					
	350	400	450	500	550	600
IBW (g)	9.96 ± 0.07	10.02 ± 0.13	10.07 ± 0.22	10.03 ± 0.05	9.98 ± 0.17	10.04 ± 0.12
WGR (%)	133.98 ± 8.77^a^	154.47 ± 9.19^ab^	168.96 ± 16.94^abc^	222.20 ± 24.66^c^	213.20 ± 9.94^bc^	204.86 ± 33.17^bc^
SGR (% )	1.52 ± 0.07^a^	1.67 ± 0.06^ab^	1.76 ± 0.12^abc^	2.08 ± 0.13^c^	2.04 ± 0.06^bc^	1.97 ± 0.l9^bc^
FCR	1.41 ± 0.01^d^	1.30 ± 0.04^c^	1.25 ± 0.04^bc^	1.07 ± 0.01^a^	1.17 ± 0.03^ab^	1.19 ± 0.04^b^
PER	2.02 ± 0.02^d^	1.93 ± 0.06^ cd^	1.78 ± 0.05^bc^	1.71 ± 0.03^b^	1.69 ± 0.02^b^	1.40 ± 0.05^a^
SR (%)	90.67 ± 3.53	92.00 ± 4.62	94.67 ± 3.53	86.67 ± 3.53	86.67 ± 3.53	82.67 ± 3.53

Values in the table are means ± SD (n = 3). Values in the same line with different superscripts indicate significant difference (*P* < 0.05).

*IBW* initial body weight, *FBW* final body weight, *WGR* weight gain rate, *SGR* specific growth rate, *FCR* feed conversion ratio, *PER* protein efficiency ratio, *SR* survival rate.

**Table 2:** Effect of dietary protein level on morphological indices of juvenile orange-spotted grouper.

Parameters	Dietary protein levels (g/kg)					
	350	400	450	500	550	600
CF (g/cm^3^)	3.28 ± 0.05^c^	3.20 ± 0.19^bc^	3.19 ± 0.01^bc^	2.99 ± 0.05^b^	2.94 ± 0.03^b^	2.67 ± 0.02^a^
HSI (%)	2.99 ± 0.05^b^	2.85 ± 0.16^ab^	2.81 ± 0.09^ab^	2.74 ± 0.02^ab^	2.70 ± 0.07^ab^	2.62 ± 0.05^a^
VSI (%)	10.33 ± 0.15^b^	10.08 ± 0.08^ab^	10.12 ± 0.13^ab^	10.02 ± 0.19^ab^	9.87 ± 0.18^ab^	9.67 ± 0.05^a^

Values in the table are means ± SD (n = 3). Values in the same line with different superscripts indicate significant difference (*P* < 0.05).

*CF* condition factor, *HSI* hepatosomatic index, *VSI* visceralsomatic index.

### Muscle and whole-body composition

The moisture and ash of whole body and muscle were not affected by dietary protein level (*P* < 0.05), as shown in Table 3. The crude protein content of whole fish and muscle increased obviously with the increase of dietary protein level (*P* < 0.05). The muscle protein content in 350 g/kg and 400 g/kg groups were significantly lower than those the feed protein level reached or exceeded 500 g/kg groups (*P* < 0.05), and the whole fish protein content in 350 g/kg group was significantly lower than those the feed protein level reached or exceeded 500 g/kg groups (*P* < 0.05). Nevertheless, the crude lipid content of whole fish and muscle reduced significantly (*P* < 0.05). The crude lipid content of whole fish in 350, 400 g/kg groups and muscle lipid content in 350, 400, 450 g/kg groups were significantly higher than those the feed protein level reached 550 g/kg groups (*P* < 0.05).

**Table 3:** Effect of dietary protein level on whole body and muscle composition of juvenile orange-spotted grouper (g/kg).

Parameters	Dietary protein levels (g/kg)					
	350	400	450	500	550	600
**Whole body**						
Moisture	705.3 ± 3.6	696.0 ± 3.7	698.4 ± 2.1	699.0 ± 2.5	706.5 ± 1.5	710.2 ± 8.4
Crude protein	565.6 ± 3.1^a^	572.4 ± 6.4^ab^	582.8 ± 2.4^b^	584.8 ± 4.1^bc^	596.9 ± 4.4^c^	610.1 ± 0.6^d^
Crude lipid	281.6 ± 0.3^c^	281.3 ± 1.9^c^	276.8 ± 0.9^bc^	276.7 ± 1.9^bc^	271.8 ± 3.6^b^	260.7 ± 1.4^a^
Ash	132.3 ± 2.4	132.9 ± 1.2	133.6 ± 0.1	133.8 ± 1.9	132.5 ± 1.5	137.0 ± 0.3
**Muscle**						
Moisture	792.3 ± 8.9	789.5 ± 4.2	779.2 ± 0.6	777.9 ± 0.2	778.4 ± 1.1	780.0 ± 0.4
Crude protein	854.0 ± 1.8^a^	858.4 ± 2.9^ab^	863.2 ± 7.6^bc^	868.0 ± 1.0^c^	868.1 ± 0.4^c^	868.6 ± 2.7^c^
Crude lipid	84.7 ± 1.4^c^	82.4 ± 1.2^c^	83.3 ± 1.5^c^	78.2 ± 4.9^bc^	73.9 ± 1.2^b^	65.8 ± 0.6^a^
Ash	60.7 ± 2.3	61.8 ± 0.7	62.7 ± 1.3	63.0 ± 1.3	64.1 ± 1.0	64.5 ± 0.7

Notes: Values in the table are means ± SD (n = 3). Values in the same line with different superscripts indicate significant difference (*P* < 0.05).

### Plasma biochemical parameters

**Figure 2:**
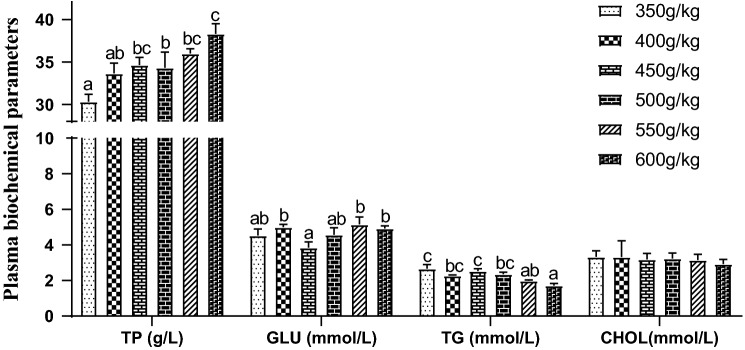
Effect of dietary protein level on plasma biochemical parameters of juvenile orange-spotted grouper. Values are means ± SD (n = 3). Different letters assigned to the bars represent significant differences using Duncan's multiple comparison (*P* < 0.05). *TP* total protein, *GLU* glucose, *TG* triglyceride, *CHOL* cholesterol.

Total plasma protein (TP) content of grouper increased gradually with the increase of feed protein level, and it was significantly lower in 350, 400 and 500 g/kg groups than in 600 g/kg group (*P* < 0.05), Fig. 2. The maximum plasma glucose in 550 g/kg group was significantly higher than that in 450 g/kg group (*P* < 0.05); at this time, there was no significant difference between other groups. With the increase of protein level, the plasma triglyceride (TG) content reduced significantly (*P* < 0.05), and 600 g/kg group was obviously lower than those in 350–500 g/kg groups (*P* < 0.05). However, feed protein level had no effect on plasma cholesterol (CHOL) (*P* > 0.05).

### Liver biochemical parameters and immune enzyme activities

**Figure 3:**
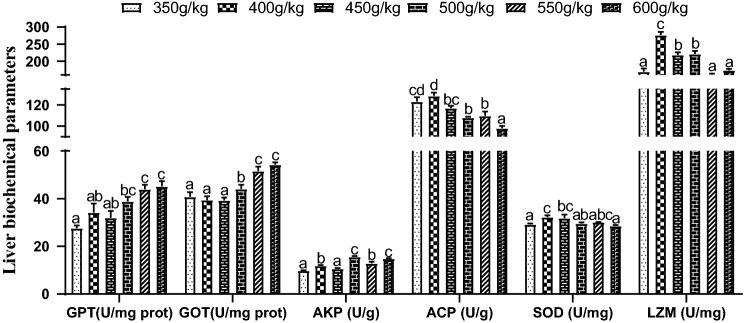
Effect of dietary protein level on liver biochemical parameters and immune enzyme activities of juvenile orange-spotted grouper. Values are means ± SD (n = 3). Different letters assigned to the bars represent significant differences using Duncan's multiple comparison (*P* < 0.05). *GPT* glutamic-pyruvic transaminase, *GOT* glutamic oxaloacetylase, *AKP* alkaline phosphatase, *ACP* acid phosphatase, *SOD* superoxide dismutase, *LZM* lysozyme.

In 350–450 g/kg groups, the glutamic-pyruvic transaminase (GPT) activities in liver were obviously lower than those in feed protein level was above 550 g/kg groups (*P* < 0.05) (Fig. 3). And the activity of glutamic oxaloacetic transaminase (GOT) in 350–500 g/kg groups was obviously lower than those in feed protein level was above 550 g/kg groups (*P* < 0.05). Meanwhile, the liver GOT activities in 350–450 g/kg groups were significantly lower than that in 500 g/kg group (*P* < 0.05). The highest activities of alkaline phosphatase (AKP), superoxide dismutase (SOD) and lysozyme (LZM) in liver appeared in 500, 400 and 400 g/kg group, respectively. AKP activity in 500 g/kg group was significantly higher than those in 350–550 g/kg groups (*P* < 0.05), the SOD activity in 400 g/kg group was obviously higher than those in 350, 500 and 600 g/kg groups (*P* < 0.05), and the LZM activity in other groups was significantly lower than that in 400 g/kg group (*P* < 0.05). When protein level was above 500 g/kg, the acid phosphatase (ACP) activity of liver was obviously higher than those in 350 and 400 g/kg groups.

### Intestine digestive enzyme activities

**Figure 4:**
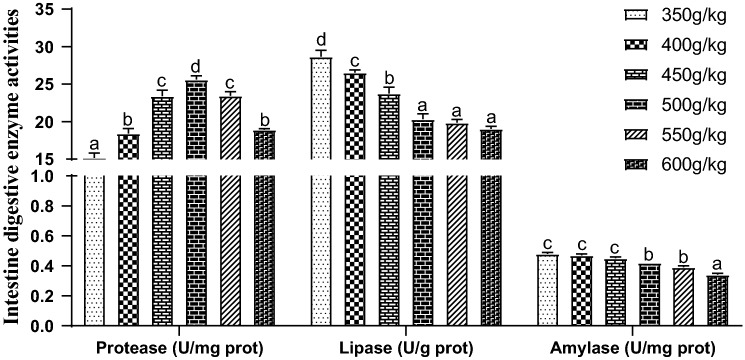
Effect of dietary protein level on intestine digestive enzyme activities of juvenile orange-spotted grouper. Values are means ± SD (n = 3). Different letters assigned to the bars represent significant differences using Duncan's multiple comparison (*P* < 0.05).

The level of feed protein significantly changed the intestine digestive enzyme activity of juvenile orange-spotted grouper (*P* < 0.05) (Fig. 4). The activities of intestine amylase and lipase decreased significantly with the increase of protein level (*P* < 0.05), and the activities of amylase and lipase in other groups were higher than those in protein level reached or exceeded 500 g/kg groups (*P* < 0.05). The protease activity in 350 g/kg group was lowest and significantly lower than those other groups, and protease activity in 500 g/kg group was highest and significantly higher than those other groups (*P* < 0.05).

## Discussion

Protein, one of the important nutrients for fish growth, is also the main substance for tissue renewal and repair^23^. Essential amino acids supplied for fish growth are all provided by feed protein, and feed protein provides some energy for organisms. Diet protein not only provides essential amino acids for fish growth, but also provides part of energy for the organism. When the diet protein content is too low, the growth of fish will be inhibited; if the protein content is too high, part of it will be used to energy metabolism, which will lead to an increase in the emission of nitrogenous substances, further resulting in the growth and development of fish caused by water environment pollution^24,25^. The present research results showed that the SGR and WGR of juvenile orange-spotted grouper increased first and then reduced with the rasing of feed protein level, which in keeping with the research of striped catfish^8^ and silver catfish (*Rhamdia quelen*)^26^, these suggested the high level of feed protein can cause the fish growth rate declines^27^. The experiment showed that the optimum dietary protein level of juvenile orange-spotted grouper was 521.84 g/kg. While other research reported that the protein requirement of juvenile orange-spotted grouper (10.7 ± 0.2 g) was 480 g/kg^22^, this may be due to experimental feed formulas, different protein sources and different digestibility of fish to various raw materials, etc. In addition, the protein requirement of juvenile grouper (*Epinephelus akaara*) (7.88 ± 0.04 g) was 508.3 g/kg^28^, which was consistent with this study. However, the optimum protein level of grow-out stage^20^ orange-spotted grouper and large-size orange-spotted grouper^21^ were 466.65 g/kg and 438.39 g/kg. It is suggested that the optimal protein requirements of the same species of fish with the same feed formula and different specifications are different. Meanwhile, this also verifies that protein requirements are inversely proportional to fish size^29^. In present test, The FCR of juvenile Epinephelus coioides decreased gradually due to the increase of dietary protein level, and the results of this study were consistent with those of previous studies^23,30^, the reduction of protein utilization rate may be caused by excessive deamination of amino acids as energy consumption^31^. In previous studies on red spotted grouper^28^ and hybrid grouper (*Epinhelus fuscoguttatus* × *E. lanceolatus*)^32^, the remarkable decrease of PER of grouper when fed high protein feed, which was consistent with the experimental results. However, there was study^30^ shown that as protein levels in the feed increase, PER incremental increased and then decreased. These results shown that when ingestion low-protein diet, the absolute intake of protein was lower, and the intake of protein mainly to keep the growth needs of fish, so as to obtain a higher protein utilization ratio; when ingestion high-protein diet, part of the excessive absolute protein will be used to maintain the energy supply required for the growth of fish, so as to reduce the protein utilization ratio^31^.

The morphological index is a momentous index that affect fish yield^33^, and also influenced by protein level^28^. The decrease of CF, VSI and HSI is due to the increase of feed protein level, which was consistent with the results of red grouper^28^, *Takifugu obersus*^30^ and Asian catfish (*Clarias batrachus*)^34^. This may have a bearing on the decreasing of diet carbohydrate content.

According to the previous experimental, the feed protein level is directly proportional to the fish protein content^35,36^, the protein intake of fish increases with the rasing of feed protein level. The present results were consistent with those conclusions, a large number of protein was ingested and digested, which can be used to repair and renew protein tissue for fish^37^, so that whole fish and muscle protein content increased. However, with the rasing of feed protein level, the whole fish and muscle crude lipid content decreased, which was consistent with the results of Wuchang Sparus macrocephalus (*Megalobrama amblycephala*)^37^. This may be due to the insufficient protein synthesis in the body when the fish feed on the low-protein level feed, which affects the synthesis of lipid metabolizing enzymes, resulting in the deposition of body lipid, resulting in the lower crude protein content and the higher crude lipid content; when that protein level of the fee reaches the requirement of fish, it can promote the synthesis of metabolic enzymes in the body, thus inhibiting the deposition of lipid in the body^38^.

Metabolic and physiological status of fish, health status and adaptability to environment of fish can be evaluated by using various plasma biochemical indicators^39^. The metabolism of carbohydrate, lipid and protein can be observed by GLU, TG and TP^40^. GLU is formed by digestion and absorption of carbohydrate in diet and decomposition of glycogen in liver and muscle. Dietary and nutritional suitability and important indexes of liver and pancreas function can be evaluated by GLU, and the evaluation of animal glucose metabolism, tissue and cell function and endocrine function can also be reflected by GLU. CHOL, the main component of cell membrane, plays a vital and indispensable role in maintaining normal physiological functions of cells. The study suggested that CHOL and TG of juvenile orange-spotted grouper decreased with the increase of dietary protein level, which was similar to other aquatic animals^41,42^. High carbohydrate content may affect the state of high TG and CHOL levels in low protein/high carbohydrate diet. Low protein/high carbohydrate diet will produce more acetyl CoA and dihydroxyacetone phosphate and provide more GLU in the process of glycolysis, at which time TG and CHOL are also produced by lipid synthesis^30^. The absorption and metabolism of protein in vivo can be evaluated by TP content in plasma^43^. The blood protein main is to maintain colloidal osmotic pressure, which has transport, immunity, tissue repair, buffer functions. The rasing of plasma protein content can improve the metabolic level and immune capacity of animals, promote nitrogen deposition and protein synthesis^44^. With the increase of dietary protein content, TP level gradually increased, suggesting that liver function was enhanced and HSI was gradually decreased.

GOT and GPT, as transaminases of protein metabolism, mainly appears in the liver, the varieties of these two enzymatic activity mirror the states of protein metabolism for fish^45^. The results of Nile tilapia^5^and gibel carp^46^ are similar to those of this experiment: the activity of GPT and GOT in low protein group is obviously lower than that in high protein group (*P* < 0.05). The protein synthesis and metabolism of organism is mainly participated by the protein in feed, so when grouper feeds low protein level grain, it has excess energy, so there is no need to decompose a large amount of protein to provide energy, because its protein synthesis in vivo is insufficient. The peak growth of fish is due to the gradual satisfaction of fish's demand for protein and the continuous improvement of dietary protein level. The main participants in protein metabolism, GOT and GPT, have increased their activities with the acceleration of protein metabolism under the optimal growth conditions.

The immune status of fish is also related to protein, because the main substance synthesized by various immune enzymes and antibodies in the body is protein^47^. Compared with terrestrial animals, the specificity immunological functioning of fish is incomplete, thus, fish mainly rely on nonspecific immunity to resist the invasion of pathogenic microorganisms. AKP, ACP, SOD and LZM are considerable indicators to evaluate immune and health status^48^. The content of AKP and ACP in liver is an important index to reflect liver metabolism. SOD is important intracellular antioxidant enzyme, its main function is to clean up unnecessary radicals in organism, and avoid damage caused by peroxidation, strengthen the defense ability of phagocytes and improve immune response. LZM is a cell non-specific immune protein, which plays a central role in immune defense process^49^. In present paper, the liver ACP, SOD and LZM activities of juvenile orange-spotted grouper upped first and then downed. This suggested that insufficient protein intake will reduce fish growth and immunity^50^; excessive protein intake will reduce protein digestibility^51^, cause intestinal diseases, and affect the immune function of the body; and the stability of antioxidant system and health require proper protein, because the formation of antioxidants and immune enzymes requires protein to provide a large number of amino acids^11^.

Digestive enzymes activity determines the ability of aquatic animals to digest and absorb nutrients, thus affecting the growth rate of aquatic animals^51^, and the digestive enzyme activity of fish depends on the feed composition^52,53^. When diet protein level rose from 350 to 500 g/kg, intestine protease activity gradually increased, and began to decrease when the protein level outstripped 500 g/kg. This suggested that diet protein level outstrips a certain limit, it will reduce the digestive capacity of fish. This is consistent with previous studies^51^. With the rasing of dietary protein level, the intestinal lipase activity about silver barb (*Puntius gonionotus* fingerlings)^54^ and striped catfish (*Pangasianodon hypophthalmus*)^8^ increased first and then reduced, while the intestine lipase activity reduced continuously in present study, which owing to different fish and diet formula. Meanwhile, with the rasing of dietary protein level, the intestinal amylase activity reduced, this probable owing to the reduction of nitrogen-free extract in diet^55^, and similar results appeared in the study of Jayant, et al.^8^.

## Materials and methods

All animal experiments were conducted strictly based on the recommendations in the ‘Guide for the Care and Use of Laboratory Animals’ set by the National Institutes of Health. We obtained permission to conduct this study from the ethics review board of the Institutional Animal Care and Use Committee (IACUC) of Guangdong Ocean University (Zhanjiang, China). All experiments were performed in accordance with relevant named guidelines and regulations. The study was carried out in compliance with the Arrive guidelines.

Six kinds of iso-lipid (124 g/kg) feeds with different protein levels (350, 400, 450, 500, 550 and 600 g/kg) were prepared. The experimental diet formula and nutrient composition were shown in Table 4. The experiment was conducted in triplicates with 25 fish (10.02 ± 0.22 g) per replicate. The diet preparation procedure, experimental procedure, sample collection, methods of analysis and statistical analysis are consistent with our previous study^20,21^, and the detailed steps are shown in the materials and methods supplementary documents.

**Table 4:** Formulation and proximate composition of the experimental diets (g/kg dry matter).

Ingredient	Dietary protein level (g/kg)					
	350	400	450	500	550	600
White fish meal	360.0	360.0	360.0	360.0	360.0	360.0
Casein	75.0	130.0	185.0	240.0	295.0	350.0
Wheat flour	183.2	183.2	183.2	183.2	183.2	183.2
α-starch	275.0	220.0	165.0	110.0	55.0	0.0
Soybean lecithin	45.0	45.0	45.0	45.0	45.0	45.0
Fish oil	45.0	45.0	45.0	45.0	45.0	45.0
Vitamin C (35%)	0.5	0.5	0.5	0.5	0.5	0.5
Choline chloride	5	5	5	5	5	5
Vitamin premix^a^	3.0	3.0	3.0	3.0	3.0	3.0
Mineral premix^a^	7.0	7.0	7.0	7.0	7.0	7.0
Attractant	1.0	1.0	1.0	1.0	1.0	1.0
Ethoxyquin	0.3	0.3	0.3	0.3	0.3	0.3
Proximate composition^b^						
Crude protein/(g/kg)	358.1	408.6	451.8	502.1	555.1	604.4
Crude lipid/(g/kg)	123.4	123.8	124.1	124.2	125.9	126.7
Ash/(g/kg)	109.3	109.6	101.2	104.9	105.2	105.2

^a^Vitamin and mineral premix were obtained from Qingdao Master Biotechnology Co, Ltd (Qingdao, China).

^b^Measured value.

The calculation formula of growth performance and morphological index is as follows:

$${\text{Weight gain rate }}\left( {{\text{WGR}}, \, \% } \right)\, = \,{1}00\, \times \,\left( {{\text{final weight}}{-\!\!-}{\text{initial weight}}} \right) \, /{\text{ initial weight}};$$

$${\text{Specific growth rate }}\left( {{\text{SGR}}, \, \% /{\text{day}}} \right)\, = \,{1}00\, \times \,(\left( {{\text{ln }}\left( {\text{final weight}} \right){-}{\text{ln }}\left( {\text{initial weight}} \right)} \right) \, /{\text{ days of experiment}};$$

$${\text{Survival rate }}\left( {{\text{SR}}, \, \% } \right)\, = \,{1}00\, \times \,\left( {{\text{total number of fish at termination }}/{\text{ total number of fish stocked}}} \right);$$

$${\text{Feed conversion ratio }}\left( {{\text{FCR}}} \right)\, = \,{\text{dry feed intake }}/{\text{ weight gain}};$$

$$\begin{gathered} {\text{Protein efficiency ratio }}\left( {{\text{PER}}} \right)\, = \,{1}00\, \times \,{\text{average weight gain }}/{\text{ average protein intake}}; \hfill \\ {\text{Condition factor }}({\text{CF}},{\text{g}}/{\text{cm}}^{{3}} )\, = \,{\text{wet weight of fish }}/{\text{ length of fish}}^{{3}} ; \hfill \\ \end{gathered}$$

$$\begin{gathered} {\text{Hepatosomatic index }}\left( {{\text{HSI}}, \, \% } \right)\, = \,{1}00\, \times \,\left( {{\text{liver wet weight }}/{\text{ body wet weight}}} \right); \hfill \\ {\text{Visceralsomatic index }}\left( {{\text{VSI}}, \, \% } \right)\, = \,{1}00\, \times \,\left( {{\text{viscera wet weight }}/{\text{ body wet weight}}} \right). \hfill \\ \end{gathered}$$

### Ethics statement

All animal experiments were conducted strictly based on the recommendations in the ‘Guide for the Care and Use of Laboratory Animals’ set by the National Institutes of Health. We obtained permission to conduct this study from the ethics review board of the Institutional Animal Care and Use Committee (IACUC) of Guangdong Ocean University (Zhanjiang, China). All experiments were performed in accordance with relevant named guidelines and regulations. The study was carried out in compliance with the Arrive guidelines.

## Conclusion

In conclusion, the growth performance and antioxidant capacity of grouper will be obviously improved at the appropriate protein level. Under the experimental conditions, the optimal protein requirement of juvenile orange-spotted grouper (10.02 ± 0.22 g) was 521.84 g/kg by using the discount model fitting with SGR as the evaluation index.

## Supplementary Information

Supplementary Information.

## Data Availability

The data that support the findings of this study are available on request from the corresponding author. The data are not publicly available due to privacy or ethical.
