# Author Correction: Optimum protein requirement of juvenile orange-spotted grouper (*Epinephelus coioides*)

**DOI:** 10.1038/s41598-021-97906-z

**Published:** 2021-09-03

**Authors:** Xiaobo Yan, Junjiang Yang, Xiaohui Dong, Beiping Tan, Shuang Zhang, Shuyan Chi, QihuiYang, Hongyu Liu, Yuanzhi Yang

**Affiliations:** 1 Laboratory of Aquatic Nutrition and Feed, College of Fisheries, Guangdong Ocean University, Zhanjiang, 524088 People’s Republic of China; 2 Aquatic Animals Precision Nutrition and High Efficiency Feed Engineering Research Center of Guangdong Province, Zhanjiang, 524088 People’s Republic of China; 3 Key Laboratory of Aquatic, Livestock and Poultry Feed Science and Technology in South China, Ministry of Agriculture, Zhanjiang, 524000 Guangdong People’s Republic of China

Correction to: *Scientific Reports* 10.1038/s41598-021-85641-4, published online 18 March 2021

The original version of this Article contained an error in Table 3 where a decimal point was missing in column “500” for crude protein parameter,

“5848 ± 4.1^bc^”

now reads:

“584.8 ± 4.1^bc^”

The original Article has been corrected.

