# Supplementary material for: Optimum protein requirement of juvenile orange-spotted grouper (Epinephelus coioides)
Source: Sci Rep. 2021 Mar 18;11:6230. doi: 10.1038/s41598-021-85641-4 (PMC7973734; doi:10.1038/s41598-021-85641-4)
Supplement: Supplementary file 1 — Supplementary Information. [file 41598_2021_85641_MOESM1_ESM.docx]

**Optimum protein requirement of juvenile orange-spotted grouper (*Epinephelus coioides*)**

Xiaobo Yan^1,2^, Junjiang Yang^1^, Xiaohui Dong^1,2,3*^, Beiping Tan^1,2,3*^, Shuang Zhang^1,2,3^, Shuyan Chi^1,2,3^, QihuiYang^1,2,3^, Hongyu Liu^1,2,3^, Yuanzhi Yang^1^

^1^Laboratory of Aquatic Nutrition and Feed, College of Fisheries, Guangdong Ocean University, Zhanjiang 524088, PR China

^2^Aquatic Animals Precision Nutrition and High Efficiency Feed Engineering Research Center of Guangdong Province, Zhanjiang 524088, PR China

^3^Key Laboratory of Aquatic, Livestock and Poultry Feed Science and Technology in South China, Ministry of Agriculture, Zhanjiang, Guangdong 524000, PR China

*Corresponding author: Xiaohui Dong, Laboratory of Aquatic Animal Nutrition and Feed, College of Fisheries, Guangdong Ocean University, Zhanjiang 524088, China. E-mail address: dongxiaohui2003@163.com;

*Corresponding author: Beiping Tan, Laboratory of Aquatic Animal Nutrition and Feed, College of Fisheries, Guangdong Ocean University, Zhanjiang 524088, China. E-mail address: [bptan@126.com](mailto:bptan@126.com)

**Materials and methods**

**Author statement confirming:**

All animal experiments were conducted strictly based on the recommendations in the ‘Guide for the Care and Use of Laboratory Animals’ set by the National Institutes of Health. We obtained permission to conduct this study from the ethics review board of the Institutional Animal Care and Use Committee (IACUC) of Guangdong Ocean University (Zhanjiang, China). All experiments were performed in accordance with relevant named guidelines and regulations. The study was carried out in compliance with the Arrive guidelines.

**Experimental diets**

With white fish meal and casein as protein source, fish oil and soybean phospholipid as lipid source, α-starch and flour as carbohydrate source, six kinds of equal lipid (124 g/kg) feeds with different protein levels were prepared. The protein levels of the feeds were 350 g/kg, 400 g/kg, 450 g/kg, 500 g/kg, 550 g/kg and 600 g/kg, respectively. The composition and nutrition level of the test feed were shown in Table 1. All ingredients were crushed through sixty mesh sieve, weighed according to the formula and thoroughly mixed using the progressive enlargement method as previously described by [Ayisi, et al. (2017)](#_ENREF_3), then added the pre-mixed fish oil and soybean lectithin, hand-rubbing small grease particles. After sieving, they are evenly mixed in the V-type vertical mixer, and a proper amount of water (30% ~ 40%) is added, which is evenly mixed. After then, the diets were processed into 3.0 mm diameter pellets strip by a twin screw extruder (F–26, South China University of Technology, Guangdong Province, China). Air-dried to about 10% moisture content, then ground and sieved to an appropriate size and stored in ziploc bags at -20 °C until use.

**Experimental procedure**

All animal experiments were conducted strictly based on the recommendations in the ‘Guide for the Care and Use of Laboratory Animals’ set by the National Institutes of Health. The animal protocols were approved by the Animal Ethics Committee of Guangdong Ocean University (Zhanjiang, China). Grouper was purchased from a grouper farm in Hainan Province. The experiment was carried out in the marine biology research base of Guangdong Ocean University (Zhanjiang, China). The juvenile orange-spotted grouper was temporarily raised with commercial feed (460 g/kg crude protein) for 2 weeks before the experiment, and then fed with experimental feed with 350 g/kg protein content for 1 week, so that grouper gradually adapted to the mixed feed and aquaculture water environment. After 24 hours of fasting, 450 juvenile orange-spotted grouper with strong physique, uniform size and average body weight of 10.02 ± 0.22 g were randomly divided into 18 tanks and fed 3.0 mm diameter feed. Each experimental feed was fed to triplicate groups of fish twice daily (08:00 and 17:00) until visual apparent satiation level and the amount of feeding ingestion were recorded. During the test, the water temperature was 28.5 - 31.5 °C, the dissolved oxygen content was more than 6 mg/L, the pH was 7.5 - 8, the salinity was 26 - 30, and the culture period was 8 weeks.

**Sample collection**

Samples were taken 24 hours after starvation. All the fish were fished out, anesthetized with eugenol (1:10000), weighed and counted to calculate the weight gain rate (WGR), specific growth rate (SGR), survival rate (SR), feed coefficient (FCR) and protein efficiency (PER). Three fish were randomly selected from each tank to measure their body weight and body length to calculate their condition factor (CF), then the liver and visceral mass were separated and weighed to calculate the hepatosomatic index (HSI) and visceralsomatic index (VSI). Seven fish were randomly selected from each tank. Blood were collected from the tail vein, placed in 1.5ml anticoagulant tube and placed at room temperature for 4 h, and then centrifuged (3500 ×g, 10 min, 4 °C). The collected plasma was stored in the refrigerator at - 80 °C for biochemical indexes. After blood collection, the back muscles were separated and packed in sealed bags and stored in -20 °C refrigerator for muscle composition analysis. At the same time, 3 fish were randomly selected for storage in -20 °C refrigerator for body composition measurement. Finally, three fish were randomly selected from each tank, and the livers and foregut tissues were dissected and stored in -80 °C refrigerator for biochemical index, immune index and digestive enzyme.

**The methods of analysis**

The calculation formula of growth performance and morphological index is as follows:

Weight gain rate (WGR, %) = 100 × (final weight - initial weight) / initial weight;

Specific growth rate (SGR, %/d) = 100 × ((ln (final weight) – ln (initial weight)) / days of experiment;

Survival rate (SR, %) = 100 × (total number of fish at termination / total number of fish stocked);

Feed conversion ratio (FCR) = dry feed intake / weight gain;

Protein efficiency ratio (PER) =100 × average weight gain / average protein intake;

Condition factor (CF, g/cm^3^) = wet weight of fish / length of fish^3^;

Hepatosomatic index (HSI, %) = 100 × (liver wet weight / body wet weight);

Visceralsomatic index (VSI, %) = 100 × (viscera wet weight / body wet weight).

Proximate analysis of the materials, experimental feed, whole fish and muscle followed the methods specified by AOAC ([Lee, 1995](#_ENREF_24)). Moisture content was determined by drying at 105 °C, crude protein was determined by multiplying nitrogen by 6.25 (Kjeltec^TM^ 8400, Denmark), crude lipid was determined by Soxhlet extraction (using petroleum ether as solvent), crude ash was determined by calcination at 550 °C in a muffle furnace, and energy by oxygen bomb energy meter (5E-1C Computer Calorimeter, Changsha Kaiyuan Instrument Co., Ltd.).

Serum biochemical indexes were determined by automatic biochemical analyzer (7020, Hitachi, Japan). Metabolic enzymes, digestive enzymes and immune enzymes were determined by the commercial kits (Nanjing Jiancheng Biotechnology Co., Ltd., Nanjing, China) and the corresponding operations were carried out according to the instructions. Lysozyme determination method refers to [Stolen, et al. (1990)](#_ENREF_40).

**Statistical analysis**

SPSS 17.0 (SPSS Inc., Chicago, IL, USA) statistical software was used to test the homogeneity of variance, and then one-way ANOVA was used to analyze the results. If there was significant difference (*P* < 0.05), Duncan's multiple comparison was used, and the statistical data was represented by mean ± standard deviation. With SGR as the evaluation index, the optimal protein requirement of juvenile orange-spotted grouper was obtained by mathematical model fitting.
